# Metamagnetism in a coordination polymer built of trimeric cobalt units and melamine

**DOI:** 10.1098/rsos.230910

**Published:** 2023-11-15

**Authors:** Ignacio Bernabé Vírseda, Arthur Mantel, Alexander Prado-Roller, Michael Eisterer, Hidetsugu Shiozawa

**Affiliations:** ^1^ J. Heyrovsky Institute of Physical Chemistry, Czech Academy of Sciences, Dolejskova 3, 182 23 Prague 8, Czech Republic; ^2^ Department of Inorganic Chemistry, University of Vienna, Währinger Straße 42, 1090 Vienna, Austria; ^3^ Atominstitut, TU Wien, Stadionallee 2, 1020 Vienna, Austria; ^4^ Faculty of Physics, University of Vienna, Boltzmanngasse 5, 1090 Vienna, Austria

**Keywords:** coordination polymer, spin trimer, metamagnetism

## Abstract

A coordination polymer of linear trimeric cobalt units and melamine has been synthesized. The magnetic isotherms of violet coloured crystals as long as 400 μm show a field-induced transition in an external field of about 2 T at temperatures approximately below 2 K. It is addressed that by assuming the coexistent positive and negative exchange between the nearest-neighbour spins in the linear trimer, this metamagnetism can be interpreted as a transition from antiferromagnetic to ferromagnetic exchange within each trimeric spin cluster. Although weak inter-cluster or inter-chain exchange to yield a long-range magnetic order is another possible and often attributed origin of metamagnetism in low-dimensional spin systems, this study demonstrates the significance of the exchange flip within each cluster in clustered spin networks.

## 1. Introduction

Since the subject ‘molecular magnetism’ started in the 1980s, growing expertise has led to the discovery of single molecule magnets (SMMs), and single-chain magnets (SCMs). Coordination chemistry provides a fruitful path to design SMMs and SCMs based on metal complexes, coordination polymers (CPs) and metal-organic frameworks (MOFs) [1].

**Figure 1.**
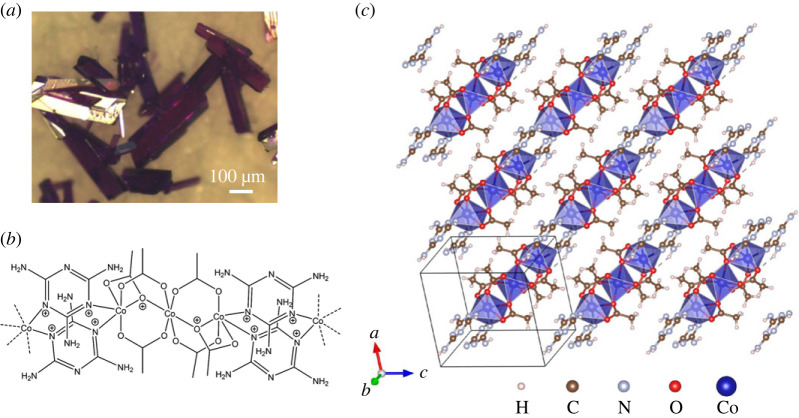
(*a*) Optical micrographs of crystals of Co_3_(CH_3_COO)_6_(C_3_H_6_N_6_)_2_. (*b*) Structural formula of the zigzag chain of Co_3_(CH_3_COO)_6_(C_3_H_6_N_6_)_2_. (*c*) Lattice structure of Co_3_(CH_3_COO)_6_(C_3_H_6_N_6_)_2_ viewed normal to the *a*–*c* plane.

In this study, we synthesize a zigzag CP of alternating trimeric cobalt–acetate units and melamine double layers, Co_3_(CH_3_COO)_6_(C_3_H_6_N_6_)_2_, as shown in figure 1. Melamine is a rich source for hydrogen bonding and despite its low solubility in organic solvents, its threefold molecular structure with pyridinic nitrogen allows it to construct molecular frameworks [2,3]. Spin trimers can be triangular or linear, and represent perfect cases for spin frustration, quantum spin liquids and multinuclear molecular magnetism. Various coordination complexes and polymers including MOFs with trimeric spin clusters were reported to exhibit a rich variety of magnetism as results of interplay between intra-trimer and inter-trimer exchange [4–11]. The magnetization isotherm of Co_3_(CH_3_COO)_6_(C_3_H_6_N_6_)_2_ exhibits a metamagnetic transition in fields about 2 T at temperatures approximately below 2 K. Some coordination complexes and polymers with trimeric transition metals were reported to exhibit field-induced metamagnetic transitions, but typically at much lower temperatures at which the thermal energy is lower than the interaction energy of interest (e.g. exchange, ligand field), or in much higher magnetic fields in which the Zeeman splitting overcomes the interaction energy [4].

Only a small number of the trimeric spin systems, metal complexes with linear cobalt dimer or trimer [7] and with triangular Mn [8], and CPs with linear cobalt trimer [6] and with triangular Fe [9], linear Mn–Ni–Mn [12], triangular Mn [10], were reported to exhibit metamagnetic transitions in similar temperature and magnetic field ranges. Similar metamagnetic properties were reported for one-dimensional CPs with Fe [9,13] or Co [14,15], and two-dimensional CPs with Co [16], Mn [17] or Ni-Cr [18], with single transition-metal nodes, and often attributed to weak interchain exchange coupling. In general, field-induced metamagnetic transitions were reported in a wide range of materials from alloys to molecular magnets. Various origins of anomalous staircase magnetism includes zero-field splitting [7], ligand-field splitting [19], spin waves [20], spin gaps or spin dimers [21,22], magnetic superlattices [23,24], structural phase transitions [25], and exchange bias. The magnetic superlattices and structural phase transitions reported in magnetic alloys and arrays of magnetic particles, and exchange bias that occurs at boundaries between soft and hard magnets, are not of molecular origin expected for CPs. The ligand-field splitting and spin waves can be observed as staircase magnetism in coordination complexes and polymers, but they are observable at much lower temperatures at which their energies exceed the thermal energy. The transition from a singlet state to a doublet state in spin gap systems requires much higher magnetic fields.

In this study, a single crystal X-ray diffraction analysis reveals that Co_3_(CH_3_COO)_6_(C_3_H_6_N_6_)_2_ is a CP in which linear trimeric cobalt-acetate units are linked via two melamine molecules. The origin of the metamagnetism can be weak inter-chain or intra-chain exchange via the aza-aromatic rings that flips the sign in a critical magnetic field, as reported elsewhere for CPs with single metal nodes. In the case of CPs with trinuclear metal nodes, intra-trimer interactions can also be considered as a possible origin of the metamagnetism. It is shown that the level crossing expected for a trimeric spin cluster with a negative exchange coupling cannot reproduce the metamagnetism in the respective temperature and field range. Instead, taking both negative and positive exchange into account, both field and temperature profiles of the magnetism can be explained. This highlights the possible importance of the exchange flip within each cluster beside the long-range magnetic order in clustered spin networks.

## 2. Results and discussion

### 2.1. Synthesis

Crystals of Co_3_(CH_3_COO)_6_(C_3_H_6_N_6_)_2_ were synthesized as follows. In total, 2 ml of 0.2 M melamine in a mixture of dimethyl sulfoxide (DMSO) and acetic acid with a volume ratio of (DMSO : acetic acid) = (95 : 5), and 2 ml of 0.1 M cobalt(II) acetate tetrahydrate in methanol were mixed and sealed in a 10 ml glass vial at room temperature, and then heated in an oven at 50°C for 24 h. The yield for the synthesis is 85±2 wt%. See the electronic supplementary material, S1 for more details. Figure 1 displays the micrographs of crystals of Co_3_(CH_3_COO)_6_(C_3_H_6_N_6_)_2_ showing the intense violet colour and edgy rhombohedral shape.

### 2.2. X-ray crystallography

A single crystal X-ray diffraction analysis of Co_3_(CH_3_COO)_6_(C_3_H_6_N_6_)_2_ reveals that it is a CP in which linear trimeric cobalt–acetate units are linked via two melamine molecules to form infinite zigzag chains along the [101] direction,^1^ as shown in figure 1.

Each cobalt atom in the trimeric unit is in an octahedral coordination geometry. The coordination sphere of the two terminal cobalt atoms (Co1) is composed of six ligands, which are two oxygen atoms of one acetate, two oxygen atoms of the other two acetate molecules, and two pyridinic nitrogen atoms of two melamine molecules. The lengths of the four Co–O bonds are in the range of 2.007−2.164 Å, and the lengths of the two Co–N bonds to the pyridinic nitrogen atoms of two melamine molecules are 2.127 and 2.397 Å. A narrow angle of 61.07° between the two adjacent Co–O bonds to the same acetate molecule leads to a largely distorted octahedral geometry of Co1.

The coordination sphere of the cobalt atom in the middle of the trimer (Co2) is composed of six oxygen atoms of six acetate molecules. The lengths of the six Co–O bonds are in the range of 2.030−2.117 Å, and the adjacent Co–O bond angles are in the range of 86.06−93.94°.

See the electronic supplementary material, S2 for more details on the structure analysis. To the best of our knowledge, this CP has not been previously reported.

### 2.3. Magnetism

**Figure 2.**
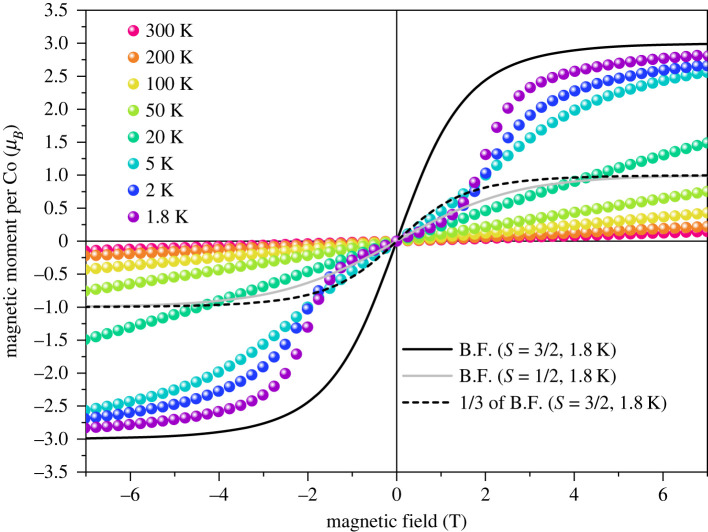
Magnetization isotherms of Co_3_(CH_3_COO)_6_(C_3_H_6_N_6_)_2_ measured at temperatures of 1.8, 2, 5, 20, 50, 100, 200 and 300 K. The Brillouin functions (B.F.) for *S* = 3/2 and 1/2 at 1.8 K are plotted as black and grey curves, respectively. The dashed curve is one-third of B.F. for *S* = 3/2 at 1.8 K.

The magnetization isotherms of Co_3_(CH_3_COO)_6_(C_3_H_6_N_6_)_2_ measured at temperatures of 2, 5, 20, 50, 100, 200 and 300 K are plotted in figure 2. At temperatures below 2 K, the staircase profile is prominent with a critical field of about 2 T. In lower fields, the isotherm follows approximately the Brillouin function for *J* = 1/2 while in high fields it approaches to 3.0 μ_*B*_, the saturation magnetism for the J=3/2 state. Assuming that the orbital magnetic moment is entirely quenched, this staircase isotherm can be interpreted as a spin transition of cobalt from *S* = 1/2 to *S* = 3/2.

#### 2.3.1. Single-ion magnetism

The electronic configuration of divalent cobalt Co(II) is [Ar] 3d^7^. The ligand field strength determines the splitting between the t_2*g*_ and e_*g*_ orbitals in an octahedral geometry. In a weak ligand field, the ground state configuration is $t_{2g}^{5}{\left(e\right)}^{2}$ resulting in a high spin state with total spin *S* = 3/2. The ground state term of this quartet state is ^4^T_1*g*_. A perfect octahedral coordination geometry O_*h*_ is hard to realize, and in most cases the octahedron is axially distorted to a tetragonal D_4*h*_ geometry [3]. An axial elongation causes a splitting of the t_2*g*_ level to the degenerated low-energy d_*xz*_ and d_*yz*_ orbitals, and the high-energy d_*xy*_ orbital. The e_*g*_ level splits into low-energy $d_{3z^{2}-r^{2}}$ and high-energy $d_{x^{2}-y^{2}}$. The corresponding term symbols for the ground and first excited states are ^4^A_2*g*_ and ^4^E_*g*_, respectively, split by energy Δ. The ^4^A_2*g*_ state further splits into two Kramers doublets, $m_{s}=\pm 1/2\;\mathrm{and}\;\pm 3/2$, as a result of spin–orbit coupling with zero field splitting (ZFS) energy *D*. The ground state is *m*_*s*_ = ±1/2 for *D* > 0 and *m*_*s*_ = ±3/2 for *D* < 0.

The spin Hamiltonian taking the zero-field splitting and the first Zeeman term into account is [26]:

2.1 $${\hat{H}}_{zfs}=D\left[{\hat{S}}_{z}^{2}-\frac{1}{3}S(S+1)\right]+g_{∥}\mu_{B}B_{z}{\hat{S}}_{z}+g_{⊥}\mu_{B}(B_{x}{\hat{S}}_{x}+B_{y}{\hat{S}}_{y}),$$

where *D* is the ZFS parameter, ${\hat{S}}_{x}$, ${\hat{S}}_{y}$ and ${\hat{S}}_{z}$ represents spin operators, S is the total spin quantum number, *B*_*x*_, *B*_*y*_ and *B*_*z*_ are the three scalar components for external magnetic field, $g_{∥}$ and *g*_⊥_ are the g-tensors in the direction parallel and perpendicular to the *z*-axis, μ_*B*_ is the Bohr magneton. The second term in the expression is the spin Zeeman term. Considering only the first-order Zeeman term, and *B*_*y*_ = *B*_*z*_ = 0, energy levels *E*_*n*_ for *S* = 3/2 are ±1/2 *gμ*_*B*_*B* − *D* for ±*m*_*S*_ = 1/2 and ±3/2 *gμ*_*B*_*B* + *D* for ±*m*_*S*_ = 3/2.

The corresponding partition function is

2.2 $$Z_{zfs}=\sum_{n}\;e^{-\beta E_{n}}=e^{\beta D}\;e^{(1/2)\beta g\mu_{B}B}+e^{\beta D}\;e^{-(1/2)\beta g\mu_{B}B}+e^{-\beta D}\;e^{(3/2)\beta g\mu_{B}B}+e^{-\beta D}\;e^{-(3/2)\beta g\mu_{B}B},$$

where *β* = 1/*k*_*B*_ *T*, and *k*_*B*_ is the Boltzmann constant. Taking only the linear Zeeman term into account, the magnetic moment per cobalt, as the first derivative of the logarithm of Helmholz free energy *F* with respect to magnetic field *B* at a given temperature, is given as

2.3 $$\begin{array}{rl} M_{zfs} & =\frac{\partial \beta^{-1}\;\ln Z_{zfs}}{\partial B}=\frac{\beta^{-1}}{Z_{zfs}}\frac{\partial Z_{zfs}}{\partial B}\\ & =\frac{g\mu_{B}}{2Z_{zfs}}[e^{\beta D}\;e^{(1/2)\beta g\mu_{B}B}-e^{\beta D}\;e^{-(1/2)\beta g\mu_{B}B}+3\;e^{-\beta D}\;e^{(3/2)\beta g\mu_{B}B}-3\;e^{-\beta D}\;e^{-(3/2)\beta g\mu_{B}B}]. \end{array}$$

**Figure 3.**
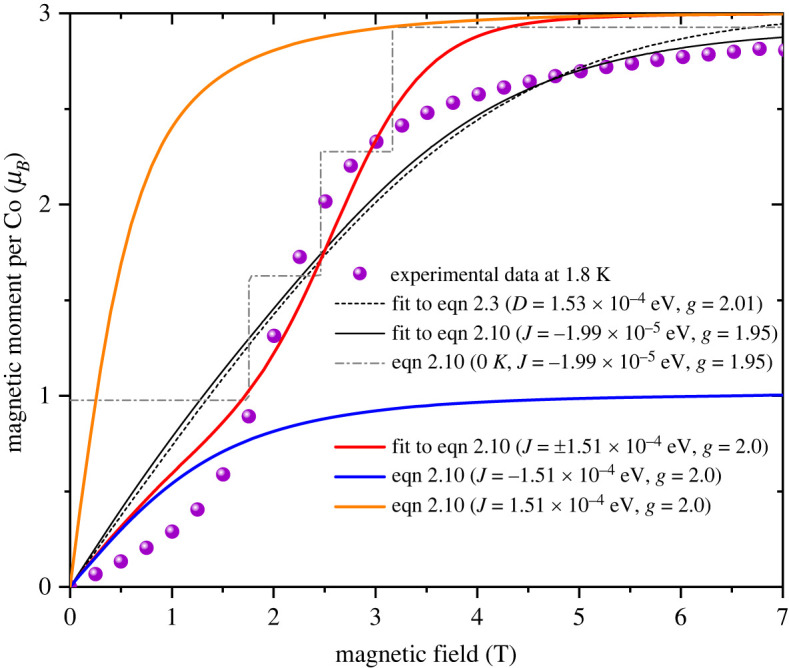
Magnetization isotherms of Co_3_(CH_3_COO)_6_(C_3_H_6_N_6_)_2_ measured at 1.8 K with the best fitting curves of equation (2.3) (dashed curve), equation (2.10) with a negative *J* (black solid curve), and the profile of equation (2.10) at 0 K (dot-dashed profile). The best fitting curve of equation (2.10) with bipolar exchange *J* = ±1.51 × 10^−4^ eV (red curve), and the profiles of equation (2.10) with only negative *J* = −1.51 × 10^−4^ eV (blue curve) and only positive *J* = 1.51 × 10^−4^ eV (orange curve) are also presented.

Figure 3 shows the magnetization isotherms of Co_3_(CH_3_COO)_6_(C_3_H_6_N_6_)_2_ at 1.8 K and the best fitting curve of equation (2.3) (dashed curve). It is apparent that the staircase magnetism observed at 1.8 K cannot be explained by the single-ion model in which the zero-field splitting becomes prominent only at much lower temperatures. The same is true even if different magnetic anisotropies for the central and terminal cobalt atoms are taken into account. Hence, the staircase magnetism with no magnetic hysteresis observed at temperatures as high as a few kelvins can be attributed to exchange coupling among cobalt spins, that can be intra-trimer or inter-trimer, or both.

#### 2.3.2. Trimeric spins

Here we consider exchange within the trimer. In this scenario, each cobalt trimer is in a triplet state (total spin *S* = 3/2) with moderate intra-trimer antiferromagnetic exchange. Inter-trimer exchange coupling is omitted, assuming that both intra- and inter-chain exchanges between trimers are too weak to be discerned in the measured conditions. We assume no single ion magnetic anisotropy, which allows bistability of both negative and positive exchange. Possible spin states of the trimer are ↓↓↓, ↑↓↓, ↓↑↓, ↓↓↑, ↑↑↓, ↑↓↑, ↓↑↑, and ↑↑↑. Considering the trimeric structure of mirror symmetry, ↓↓↓, ↓↑↓, ↑↓↑, ↑↑↑ are the most likely states. The spin Hamiltonian for a trimeric spin system is

2.4 $${\hat{H}}_{\mathrm{timer}}=-2J_{12}{\hat{S}}_{1}{\hat{S}}_{2}-2J_{12}{\hat{S}}_{2}{\hat{S}}_{3}-2J_{31}{\hat{S}}_{3}{\hat{S}}_{1},$$

where *J*_*ij*_ is the exchange integral between the *i*th and *j*th spins, and ${\hat{S}}_{i}$ is the spin operator for the *i*th spin. Assuming *J*_12_ = *J*_23_ = *J* and *J*_31_ = *αJ*, the spin Hamiltonian is reduced to

2.5 $${\hat{H}}_{\mathrm{timer}}=-2J[{\hat{S}}_{1}{\hat{S}}_{2}+{\hat{S}}_{2}{\hat{S}}_{3}+\alpha {\hat{S}}_{3}{\hat{S}}_{1}].$$

Introducing ${\hat{S}}_{t}={\hat{S}}_{1}+{\hat{S}}_{2}+{\hat{S}}_{3}$ and ${\hat{S}}_{31}={\hat{S}}_{3}+{\hat{S}}_{1}$ [27], and taking the first Zeeman term into account, we obtain

2.6 $${\hat{H}}_{\mathrm{trimer}}=-J[{\hat{S}}_{t}^{2}+(1-\alpha ){\hat{S}}_{31}^{2}-\alpha {\hat{S}}_{1}^{2}-{\hat{S}}_{2}^{2}-\alpha {\hat{S}}_{3}^{2}]+g\mu_{B}B{\hat{m}}_{t}.$$

Hence, the total energy is given by

2.7 $$\begin{array}{rl} E(S_{t},S_{31},m_{t}) & =E_{0}(S_{t},S_{31})+g\mu_{B}Bm_{t}\\ & =-2J[S_{t}(S_{t}+1)-(1-\alpha )S_{31}(S_{31}+1)-(1-2\alpha )S(S+1)]+g\mu_{B}Bm_{t}, \end{array}$$

where *S*(*S* + 1) is the eigenvalue of ${\hat{S}}_{i}^{2}$ (i = 1,2,3), *S*_31_ = 2*S*, 2*S* − 1, …, 0, and *S*_*t*_ = *S*_31_ + *S*, *S*_31_ + *S* − 1, …, |*S*_31_ − *S*|, and *m*_*t*_ = −*S*_*t*_, − *S*_*t*_ + 1, …, *S*_*t*_ − 1, *S*_*t*_ is the eigenvalue of the ${\hat{m}}_{t}.$

The corresponding partition function is

2.8 $$Z_{\mathrm{trimer}}=\sum_{S_{31},S_{t},m_{t}}\;e^{-\beta E_{0}(S_{t},S_{31})+g\mu_{B}Bm_{t}}=\sum_{S_{t},S_{31}}\;e^{-\beta E_{0}(S_{t},S_{31})}\sum_{m_{t}=-S_{t}}^{S_{t}}\;e^{-\beta g\mu_{B}Bm_{t}}.$$

Using formula $\sum_{n=0}^{N-1}r^{n}=(1-r^{N})/(1-r)$ results in

2.9 $$\begin{array}{rl} Z_{\mathrm{trimer}} & =\sum_{S_{t},S_{31}}\;e^{-\beta E_{0}(S_{t},S_{31})}\frac{e^{\beta g\mu_{B}(S_{t}+(1/2))B}-e^{-\beta g\mu_{B}(S_{t}+(1/2))B}}{e^{(1/2)\beta g\mu_{B}B}-e^{-(1/2)\beta g\mu_{B}B}}\\ & =\sum_{S_{t},S_{31}}\;e^{-\beta E_{0}(S_{t},S_{31})}\frac{\sinh[\beta g\mu_{B}(S_{t}+(1/2))B]}{\sinh[(1/2)\beta g\mu_{B}B]}. \end{array}$$

Then, the magnetic moment of the trimer is

2.10 $$\begin{array}{rl} & M_{\mathrm{trimer}}=\frac{\beta^{-1}}{Z_{\mathrm{trimer}}}\frac{\partial Z_{\mathrm{trimer}}}{\partial B}=\frac{g\mu_{B}}{Z_{\mathrm{trimer}}}\sum_{S_{t},S_{31}}\;e^{-\beta E_{0}(S_{t},S_{31})}\\ & \cdot \frac{\left(S_{t}+(1/2))\cosh[\beta g\mu_{B}(S_{t}+(1/2))B]\;\sinh[(1/2)\beta g\mu_{B}B]-(1/2)\sinh[\beta g\mu_{B}(S_{t}+(1/2))B]\cosh[(1/2)\beta g\mu_{B}B\right]}{\sinh^{2}[(1/2)\beta g\mu_{B}B]}. \end{array}$$

For a trimer of Co(II) ions, *S* = 3/2, the allowed *S*_*t*_ values are *S*_*t*_ = 3/2 for *S*_31_ = 0, *S*_*t*_ = 1/2, 3/2, 5/2 for *S*_31_ = 1, *S*_*t*_ = 1/2, 3/2, 5/2, 7/2 for *S*_31_ = 2 and *S*_*t*_ = 1/2, 3/2, 5/2, 7/2, 9/2 for *S*_31_ = 3. Plugging these combinations of *S*_*t*_ and *S*_31_ into equation (2.10), we get the magnetization per cobalt *M*_Co_ = (1/3)*M*_trimer_ for given *B*, *T*, *J*, *α* and *g*. Assuming that *J*_13_ is negligible, i.e. *α* = 0, to avoid over-parametrization, the magnetization isotherm can be fitted with equation (2.10). The best fit is obtained with *J* = −1.99 × 10^−5^ eV (−0.161 cm^−1^) and *g* = 1.95, the black solid curve in figure 3. The fitting curve does not show any stepwise feature that corresponds to the level crossing. The grey dot-dashed profile is the calculated magnetization isotherm at 0 K, exhibiting the three crossing points, (*S*_*t*_ = 3/2, *S*_31_ = 3, *m*_*t*_ = −(3/2)) → (*S*_*t*_ = 5/2, *S*_31_ = 3, *m*_*t*_ = −(5/2)), (*S*_*t*_ = 5/2, *S*_31_ = 3, *m*_*t*_ = −(52)) → (*S*_*t*_ = 7/2, *S*_31_ = 3, *m*_*t*_ = −(7/2)) and (*S*_*t*_ = 7/2, *S*_31_ = 3, *m*_*t*_ = −(7/2)) → (*S*_*t*_ = 9/2, *S*_31_ = 3, *m*_*t*_ = −(9/2)). Again, the model cannot explain the experimental data as the thermal energy at 1.8 K exceeds the Zeeman splitting in the field range where the level crossing occurs.

**Figure 4.**
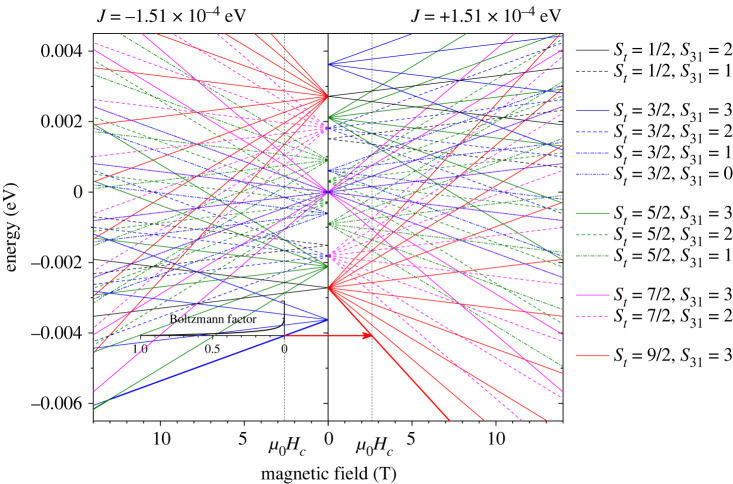
The energy levels *E*(*S*_*t*_, *S*_31_, *m*_*t*_) calculated from equation (2.7) for *J* = −1.51 × 10^−4^ eV (left panel) and *J* = 1.51 × 10^−4^ eV (right panel). The lowest energy level switches from negative exchange state (*S*_*t*_ = 3/2, *S*_31_ = 3, *m*_*t*_ = −(3/2) in the left panel) to the positive exchange state (*S*_*t*_ = 9/2, *S*_31_ = 3, *m*_*t*_ = −(9/2) in the right panel) as the field reaches critical field μ_0_*H*_*c*_.

Next, we assume that both negative and positive exchanges coexist, and all levels are occupied according to the Boltzmann distribution. In practice, the summation in equations (2.9) and (2.10) is done for a value of *E*(*S*_*t*_, *S*_31_) with positive *J* and that for negative *J* for each given *S*_*t*_ and *S*_31_ pair. Figure 4 shows *E*(*S*_*t*_, *S*_31_, *m*_*t*_) plotted against the magnetic field with a negative exchange energy of −1.51 × 10^−4^ eV (−1.22 cm^−1^) (left panel) and a positive exchange energy of 1.51 × 10^−4^ eV (1.22 cm^−1^) (right panel). *E*(*S*_*t*_, *S*_31_, *m*_*t*_) for a given combination of *S*_*t*_ and *S*_31_ with different *m*_*t*_ are plotted in the same colour.

**Figure 5.**
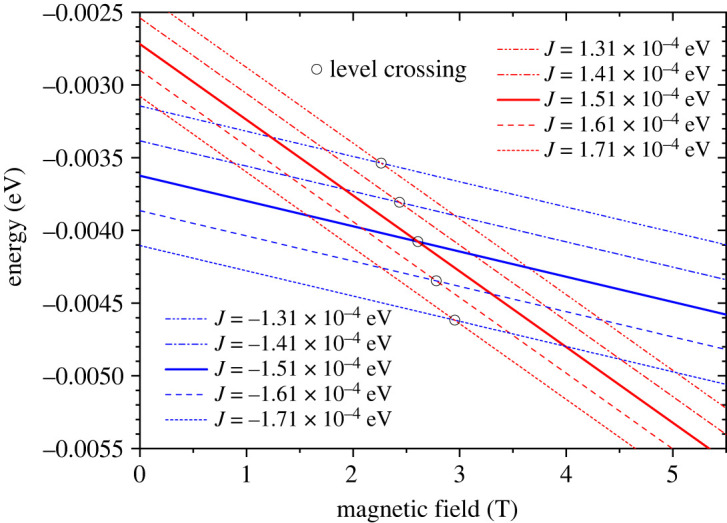
The level crossing points (open circles) between *E*(*S*_*t*_ = 3/2, *S*_31_ = 3, *m*_*t*_ = −3/2) with negative exchange (blue) and *E*(*S*_*t*_ = 9/2, *S*_31_ = 3, *m*_*t*_ = −9/2) with positive exchange (red) as a function of exchange energy ranging from ±1.31 × 10^−4^ eV to ±1.71 × 10^−4^ eV.

Importantly, the lowest energy level switches from the negative exchange state (*S*_*t*_ = 3/2, *S*_31_ = 3, *m*_*t*_ = −(3/2)) in the left panel to the positive exchange state (*S*_*t*_ = 9/2, *S*_31_ = 3, *m*_*t*_ = −(9/2)) in the right panel as the field reaches the critical field μ_0_*H*_*c*_. This transition field depends on the magnitude of exchange coupling *J*. Figure 5 shows the level crossing with different exchange energies from 1.31 × 10^−4^ eV to 1.71 × 10^−4^ eV. The best fit of equation (2.10) to the experimental magnetic isotherm is obtained with *J* = ±1.51 × 10^−4^ eV (±1.22 cm^−1^) (red solid curve in figure 3). It finally reproduces the step feature near the transition field. The exchange constant is one order greater than *J* = −1.99 × 10^−5^ eV (−0.161 cm^−1^) derived taking only the negative exchange into account. This leads to the level crossing among the negative *J* states to occur in much higher fields (the first crossing occurs in 13 T as seen in the left panel in figure 4) and hence, the same lowest negative *J* level contributes most to the net magnetism in fields below *μ*_0_*H*_*c*_.

Note that the theoretical curve does not match the experimental data in the following two respects. First, in fields below the transition field *μ*_0_*H*_*c*_, the experimental magnetic moments are smaller than the fitting curve that follows the magnetism of the negative exchange state (blue curve) saturating at about 1.0 *μ*_*B*_. The reduced magnetic moment can be owing to the ZFS. A possible positive ZFS results in the magnetic moment dominated by the Kramers doublet in low fields. Secondly, the theoretical curve saturates at about 3.0 *μ*_*B*_ in higher fields, as expected for the S=3/2 state, while the experimental data are slower to saturate or shows a lower saturation magnetism. This can be owing to the orbital magnetic moment, g-factor or asymmetric exchange or a combination of them. The linear increase of the magnetism in fields above 4 T is indicative of asymmetric exchange that is expected considering the ligand structure of three cobalt atoms in the trimer.

Exchange energies for some cobalt trimer systems were evaluated previously by fitting the temperature dependence of the magnetic susceptibility. A mean field approach taking both intra-trimer and inter-trimer exchange into account elucidated the dominant intra-trimer exchange and much weaker inter-trimer exchange in both metal complexes [28,29] and CPs [6,30], which is in line with our approach considering only the intra-trimer exchange. Exchange energies between adjacent cobalt atoms in the linear trimer reported for metal complexes are positive and in the range from 0.65 to 11.8 cm^−1^ [7,28,29,31]. On the contrary, those for CPs are negative and in the range from 0.4 to 4.87 cm^−1^ [5,6,30]. The magnitude of the exchange energy estimated in this study, i.e. ±1.51 × 10^−4^ eV (±1.22 cm^−1^), is in accordance with the energies reported for the other cobalt trimer systems mentioned above.

**Figure 6.**
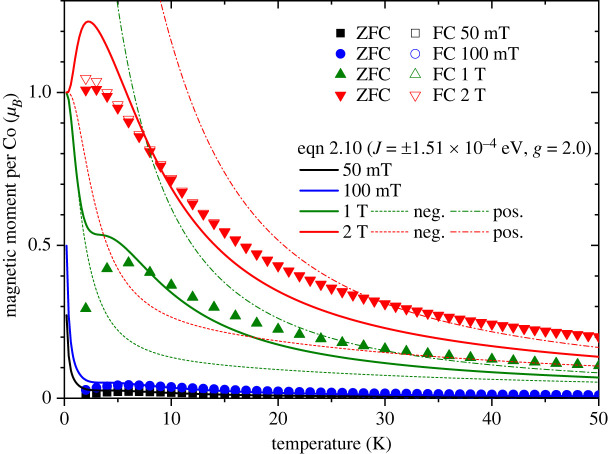
Temperature dependence of the magnetic moment per cobalt atom for Co_3_(CH_3_COO)_6_(C_3_H_6_N_6_)_2_ measured in fields of 50 mT (black rectangles), 100 mT (blue circles), 1 T (green triangles) and 2 T (red inverted triangles). The solid and open symbols correspond to the data after zero-field cooling (ZFC) and after field cooling (FC), respectively. The solid curves are equation (2.10) for *J* = ±1.51 × 10^−4^ eV. For 1 and 2 T, the temperature profiles calculated from equation (2.10) with only negative *J* = −1.51 × 10^−4^ eV (neg.) and only positive *J* = 1.51 × 10^−4^ eV (pos.) are also plotted as dashed and dot-dashed curves, respectively.

Figure 6 shows the temperature dependence of the magnetic moment per cobalt for Co_3_(CH_3_COO)_6_(C_3_H_6_N_6_)_2_ measured in 50 mT, 100 mT, 1 T and 2 T after zero-field cooling and field cooling. The solid curves are the corresponding theoretical data for bipolar exchange *J* = ±1.51 × 10^−4^ eV. The theoretical curve reproduces the main features of the experimental profiles, specifically, the peak shifting to a lower temperature as the field increases from 1 T to 2 T. The dashed and dot-dashed curves for 1 T and 2 T are calculated from equation (2.10) with only negative *J* = −1.51 × 10^−4^ eV (neg.) and only positive *J* = 1.51 × 10^−4^ eV (pos.). In fields well below the transition field *μ*_0_*H*_*c*_, the four negative *J* states of *S*_*t*_ = 3/2, *S*_31_ = 3 are at lower energies than the positive *J* states of *S*_*t*_ = 9/2, *S*_31_ = 3, see figure 4. At low enough temperatures, the four negative *J* states of *S*_*t*_ = 3/2, *S*_31_ = 3 dictate the net magnetic moment (the Boltzmann distribution at 1.8 K in the critical field *μ*_0_*H*_*c*_ is plotted in the left panel). As the temperature is raised, the positive *J* states of *S*_*t*_ = 9/2, *S*_31_ = 3 start to be occupied, that increases the net magnetism. This leads to the peak observed in the temperature dependence of the magnetic moment in 1 T and 2 T. See the electronic supplementary material, S1 for fitting of equation (2.10) to the temperature dependence of the magnetic moment measured in fields well above and below the transition field, as well as the AC susceptibility.

A quantitative analysis of the data requires a more elaborate model taking the crystal field, orbital magnetic moment, spin–orbit coupling and asymmetric exchange into account. Our model is, however, satisfactory within the scope of this study that is to seek the origin of metamagnetism within each individual cluster.

## 3. Conclusion

Single crystals of Co(II) CP Co_3_(CH_3_COO)_6_(C_3_H_6_N_6_)_2_ have been synthesized. Linear trimeric cobalt units linked by two melamine molecules offer a model case in which molecular magnetism of clustered spins in a coordination network can be studied. The magnetic isotherm of Co_3_(CH_3_COO)_6_(C_3_H_6_N_6_)_2_ measured at temperatures below 2 K shows a field-induced transition in a magnetic field about 2 T. It is addressed that taking the bipolar intra-trimer nearest-neighbour exchange into account, this metamagnetism can be attributed to the properties of exchange-coupled spins within each trimer. This study addresses the importance of a spin flip within linear spin trimers, that paves the way for a more quantitative analysis taking both intra-cluster and inter-cluster exchange into account.

## Data Availability

The data are provided in the electronic supplementary material [32].

## Footnotes

1 CCDC 2215206 contains the supplementary crystallographic data for this paper. These data can be obtained free of charge from The Cambridge Crystallographic Data Centre via www.ccdc.cam.ac.uk/structures.
